# Editorial: The relationship between health and environment under the lens of climate change: insights for policy makers

**DOI:** 10.3389/fpubh.2025.1548553

**Published:** 2025-02-04

**Authors:** Greta Colombi, Paolo Vineis

**Affiliations:** ^1^Institute of Management, Sant'Anna School of Advanced Studies, Pisa, Italy; ^2^MRC Centre for Environment and Health, Imperial College, London, United Kingdom

**Keywords:** public health, climate change, mitigation, decision making, inequalities, adaptation, policy

## Introduction

The temperature of the atmosphere continues to rise as an effect of climate change. According to WMO (World Meteorological Organization) models, there is 80% likelihood that global warming will approach 1.5°C above pre-industrial levels in the next few years, with severe direct and indirect impacts on human health (1). Addressing the climate-health relationship is therefore both highly relevant and urgent. This editorial aims to present a collection of studies that offer a comprehensive perspective on existing and emerging health issues related to climate change, while providing some insights for policymakers to guide future actions.

The effects on human health are manifold, affecting physical, emotional, and psychological wellbeing. Limited to the immediate ones, Hurricane Helene caused at least 130 deaths in the U.S., the flooding of Valencia at least 219, and the 2023 heat wave killed 47,000 people in Europe (in excess of what was expected) (2). These are just a few recent examples limited to rich countries, to which direct and catastrophic effects in Low and Middle Income Countries (LMICs) must be added, plus the indirect effects such as increased risks of infectious diseases and damage to agriculture and food production. Although attributing individual episodes (floods, hurricanes) to climate change is difficult, their sequence and intensity globally indicate that climate change is having a major impact. An international network called World Weather Attribution (WWA), which aims to establish causal links between climate change and catastrophic events, calculated that in the U.S., during Hurricane Helene, the observed precipitation was 70% and winds 150% more likely to occur as a result of climate change (3).

The World Health Organization (WHO) estimates that between 2030 and 2050, climate change could result in an additional 250,000 deaths annually (4) due to malnutrition, malaria, diarrhea, and heat stress, disproportionately affecting poorer nations with limited healthcare resources to tackle emerging environmental challenges. Air quality, closely tied to the climate crisis, represents another major global health risk. Air Pollution, exacerbated by greenhouse gas emissions and climate change, is responsible for ~6.7 million premature deaths annually (WHO), with vulnerable populations, such as children and older adults, being particularly at risk (5).

The environmental crisis is not only a physical health issue but also profoundly affects mental health. Recent studies show that people are experiencing complex psychological responses, including depression, anxiety, stress, and insomnia (6).

The human, social and economic costs of these phenomena are very high, and in the medium to long term they will outweigh the costs of mitigation, i.e., of reducing greenhouse gas emissions. In the United States alone, the number of disasters costing more than $1 billion each has increased from six in 2002 to 18 in 2022 and 28 in 2023 (7).

## The exacerbation of climate change and the need to discuss a strategy for global public health

The problem touches all generations but especially the youngest and future generations, creating severe inter-generational inequality. With each increase in global warming, regional climate changes, even radical ones, become more widespread and pronounced. This is a legacy we leave to future generations.

However, the Research Topic is not only one of inter-generational justice but also intra-generational justice. Although climate change has global coverage, its impacts are not evenly distributed. The effects of rising temperatures, extreme weather events, and biodiversity loss, combined with environmental degradation and increasing pollution, do not affect everyone equally. Those who have contributed the least to creating the problem, such as Indigenous communities, vulnerable populations, and countries in the Global South, are often the ones bearing the most severe consequences, exacerbating disparities in wellbeing and health.

The continuing emergencies we are witnessing therefore highlight the urgency of adequate prevention, given the many casualties, destruction of property and waste of public money. Prevention can be done by acting on the causes (mitigation) and defending populations against the effects (adaptation). As argued by Butler in this Research Topic of Frontiers in Public Health, we need bold new policies if we hope to avoid the collapse of human civilization, addressing both the mitigation and adaptation of climate change's effects on health and wellbeing. These policies must rely on a renewed way of doing science—one that does not shy away from difficult discussions, takes courageous positions, and is grounded in rigorous scientific methods and data.

## The obstacles to policy decisions

While discussions on private sector mobilization and global political efforts to implement mitigation measures have been ongoing for years, the recent Emissions Gap Report 2024 by UNEP has documented yet another record high in greenhouse gas emissions: in 2023, emissions reached 57.1 gigatonnes of CO_2_ equivalent, marking a 1.3% increase compared to the previous year (8).

Despite the efforts and global awareness, the data reveal a stark gap between the ambitions of the Paris Agreement and concrete action, with emissions continuing to rise globally. The achievement of the Sustainable Development Goals (SDGs) set out in the 2030 Agenda is also lagging behind. Recent analyses indicate slow progress (only in 17% of the overall SDGs), and in some cases, outright regression compared to 2015 levels (9).

Consideration must be given to what are current obstacles to global action: the export of emissions, the failure to help fossil fuel-dependent economies, the weakness of international agreements, and the costs of adaptation and mitigation strategies. The international community has attempted to address these challenges through regulatory and financial mechanisms such as the Loss and Damage funds and the Kunming-Montreal Global Biodiversity Framework. These agreements represent a step toward addressing the environmental crisis equitably and inclusively, by providing technical and financial support to the most vulnerable countries. However, available funding remains woefully insufficient compared to the scale of the needs, with an estimated gap amounting to billions of dollars.

It seems to us that at the moment the public discussion on mitigation stagnates and it is difficult to identify a shared strategy. In this context, we believe health is a central issue. While the discussion on the relationship between health and climate change gained notable attention at COP28 in Dubai, these considerations remain marginal in political discourse, corporate agendas, and public awareness. A fundamental shift in perspective is urgently needed. Health must be brought to the center of the climate conversation. As Kate Raworth reminds us, we cannot speak of a “safe and just operating space” without recognizing the inalienable right to health (10).

## The need for strategic and inclusive solutions

One of the main challenges in policy decision-making regarding health consequences of climate change lies in the uncertainty about which solutions are most effective and cost-efficient. In this sense, the calls for grants of the Wellcome Trust and ESRC in the UK, which aim to produce systematic reviews of the scientific literature on mitigation solutions, including taking into account co-benefits and costs, are crucial. A previous, partial attempt has been the Global Calculator, including incorporation of co-benefits for health (11).

While climate is a global phenomenon, its effects and impacts are heavily influenced by individual discriminatory factors, as well as sociocultural, infrastructural, and territorial vulnerabilities. For this reason, policies should: (a) be based on heterogeneous, representative and up-to date data; (b) be tailored to territorial needs rather than standardized; (c) address and combat discriminatory factors and inequalities; (d) fight cognitive biases that perpetuate unfair and unequal treatments.

Therefore, there is an urgent need to develop practical, context-specific solutions. For instance, Kennedy et al. in this Research Topic developed a method based on eco-social approaches to health to implement climate adaptation and mitigation strategies on Cortes Island, a remote area in British Columbia, Canada. Their planning was informed by community-identified needs and preferences, demonstrating the importance of integrated, solution-oriented practices grounded in the real necessities and characteristics of the territory.

A complementary approach is proposed by Katapally and Bhawra, who emphasize the use of citizen-driven big data to address the challenges of climate change. Their approach integrates accurate, timely, and multisectoral data by leveraging the widespread use of digital platforms and devices. Such data can support health systems to predict and prevent global health crises, enabling rapid responses to emerging risks and providing real-time support to citizens.

In this context, it is particularly relevant to adopt non-discriminatory approaches that respect and integrate the cultures of ethnic minorities and Indigenous peoples. These communities maintain a deeply rooted relationship with nature, consolidated within socio-cultural systems that remain intrinsically tied to it for religious, identity, economic, and survival reasons. According to recent estimations (12), in the last 10 years (between 2010 and 2020) forests—upon which roughly one-third of humanity, including many Indigenous communities, directly depends (13)—are disappearing at a rate of 4.7 million hectares per year. This loss not only threatens food and water security but also erodes the cultural identity and stability of communities. Similar challenges confront Aboriginal communities in Australia, where the increasing frequency of natural disasters, such as bushfires, has caused significant destruction. As the resources upon which these populations rely become increasingly endangered by environmental degradation, it is crucial to understand how this affects their livelihoods, psychological wellbeing, and mental health, in order to identify culturally sensitive solutions and political strategies, as suggested by Upward et al..

From a research perspective, it is essential to deepen our understanding of the relationship between external environmental factors and internal homeostasis, by financing researches to study the effects of exposure to physical, chemical, and biological stressors related to climate change on the exposome and the resulting negative health outcomes, as proposed by Robert Barouki.

However, policy makers cannot ignore a major issue in the discussion. Addressing climate change through policy also requires, as Butler highlights, fostering “nuanced, mutually respectful discussions about population and consumption.” Saraswati et al. provide essential evidence on the relationships between human population growth, environmental integrity, human prosperity, and climate change. This evidence underscores the risks of environmental degradation and the intensifying effects of climate change as populations grow, which should be considered when planning long-term solutions. These solutions should prioritize ethical family planning, women's empowerment, education, child health, and food security.

While improving data collection and accuracy, and developing tailored solutions to effectively and inclusively address the consequences of climate change, policymakers should also prioritize reducing the ecological footprint of the healthcare sector, a significant contributor to greenhouse gas emissions. Addressing this challenge requires a reimagining of healthcare processes through the adoption of innovative technologies and approaches. Policymakers should promote the application of selection criteria for healthcare procedures that consider environmental impacts alongside patient health priorities. In this context, Sack et al. demonstrated that environmentally-extended input–output analysis (EEIO) can serve as a valuable decision-making tool in the healthcare sector. Their findings suggest that the stenting pathway for stable coronary disease is preferable to coronary surgery from an environmental standpoint, as it significantly reduces the associated carbon footprint. Similarly, Usher et al. highlight the transformative potential of virtual healthcare and health education. Services such as telehealth and virtual education programs, which saw increased use and satisfaction during the COVID-19 pandemic, provide an effective means of reducing emissions associated with in-person care and education delivery. These tools have the potential to play a pivotal role in the transition toward a net carbon-zero future in the health care sector. Clear policy direction is needed to guide healthcare operators in understanding the extent to which such solutions can be applied and in which contexts, ensuring that decisions are not left solely to individual initiative or discretion.

The multiple and multidisciplinary challenges that climate change poses to health demand a paradigm shift in vision, tools, and approaches. As highlighted by Leonardi et al. (a) to successfully support the transition of human societies toward ecological sustainability, it is vital to empower practitioners across all disciplines relevant to public health. Leonardi et al. (b) advocate that supporting and transforming the education and training of current and future generations of practitioners and decision-makers is a crucial step to be implemented, fostering collaboration across countries and disciplines.

In conclusion, this Research Topic sheds light on a theme that, despite being widely discussed in scientific literature, struggles to gain a central place in international negotiations. What is required is a cultural shift, a transformation in perspective and approach—not only from healthcare workers and decision-makers but from society as a whole. The monumental and imminent challenges posed by climate change can only be faced together, through collective commitment, shared responsibility, and decisive action.
